# *Neospora caninum* Infection Triggers S-phase Arrest and Alters Nuclear Characteristics in Primary Bovine Endothelial Host Cells

**DOI:** 10.3389/fcell.2022.946335

**Published:** 2022-08-05

**Authors:** Zahady D. Velásquez, Lisbeth Rojas-Barón, Camilo Larrazabal, Marcelo Salierno, Ulrich Gärtner, Learta Pervizaj-Oruqaj, Susanne Herold, Carlos Hermosilla, Anja Taubert

**Affiliations:** ^1^ Institute of Parasitology, Biomedical Research Center Seltersberg, Justus Liebig University Giessen, Giessen, Germany; ^2^ Centre for Developmental Neurobiology, MRC Centre for Neurodevelopmental Disorders, King’s College London, London, United Kingdom; ^3^ Institute of Anatomy and Cell Biology, Justus Liebig University Giessen, Giessen, Germany; ^4^ Department of Medicine V Internal Medicine Infectious Diseases and Infection Control Universities of Giessen and Marburg Lung Center (UGMLC) Member of the German Center for Lung Research (DZL) Justus-Liebig University Giessen, Giessen, Germany; ^5^ Institute for Lung Health (ILH), Giessen, Germany; ^6^ Excellence Cluster Cardipulmonary Institute (CPI), Giessen, Germany

**Keywords:** *Neospora caninum*, apicomplexan parasites, cell cycle arrest, nuclear lamina, actin-cap

## Abstract

*Neospora caninum* represents a major cause of abortive disease in bovines and small ruminants worldwide. As a typical obligate intracellular apicomplexan parasite, *N. caninum* needs to modulate its host cell for successful replication. In the current study, we focused on parasite-driven interference with host cell cycle progression. By performing DNA content-based cell cycle phase analyses in *N. caninum*-infected primary bovine umbilical vein endothelial cells (BUVEC), a parasite-driven S-phase arrest was detected at both 24 and 32 h p. i., being paralleled by fewer host cells experiencing the G0/G1 cell cycle phase. When analyzing S-subphases, proliferation cell nuclear antigen (per PCNA)-based experiments showed a reduced population of BUVEC in the late S-phase. Analyses on key molecules of cell cycle regulation documented a significant alteration of cyclin A2 and cyclin B1 abundance in *N. caninum*-infected host endothelial cells, thereby confirming irregularities in the S-phase and S-to-G2/M-phase transition. In line with cell cycle alterations, general nuclear parameters revealed smaller nuclear sizes and morphological abnormalities of BUVEC nuclei within the *N. caninum*-infected host cell layer. The latter observations were also confirmed by transmission electron microscopy (TEM) and by analyses of lamin B1 as a marker of nuclear lamina, which illustrated an inhomogeneous nuclear lamin B1 distribution, nuclear foldings, and invaginations, thereby reflecting nuclear misshaping. Interestingly, the latter finding applied to both non-infected and infected host cells within parasitized BUVEC layer. Additionally, actin detection indicated alterations in the perinuclear actin cap formation since typical nucleo-transversal filaments were consistently lacking in *N. caninum*-infected BUVEC, as also documented by significantly decreased actin-related intensities in the perinuclear region. These data indicate that *N. caninum* indeed alters host cell cycle progression and severely affects the host cell nuclear phenotype in primary bovine endothelial host cells. In summary, these findings add novel data on the complex *N. caninum*-specific modulation of host cell and nucleus, thereby demonstrating clear differences in cell cycle progression modulation driven by other closely related apicomplexans like *Toxoplasma gondii* and *Besnotia besnoiti*.

## Graphical Abstract

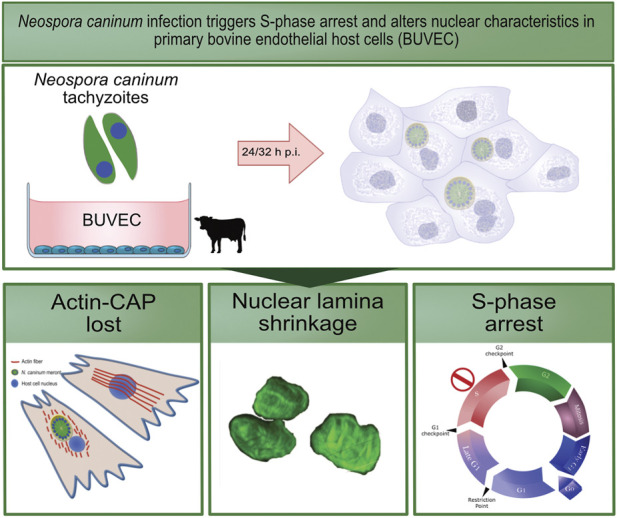

## Introduction

*Neospora caninum* is an obligate intracellular protozoan parasite belonging to the phylum Alveolata (subphylum Apicomplexa) and causing neosporosis in a wide range of warm-blooded mammals, including domestic and wild animals (Dubey et al., 2007). Neosporosis is a worldwide emerging parasitosis and is usually related to reproductive disorders (i. e., infertility, abortion, and neonatal mortality), particularly in cattle, small ruminants, and dogs (Dubey et al., 2007). Consistently, neosporosis represents a major cause of bovine abortion, resulting in high losses of over a billion dollars in the cattle industry worldwide (Dubey et al., 2007; Goodswen et al., 2013). The mode of transmission in cattle is mainly vertical by fast replicating *N. caninum* tachyzoites, causing a high prevalence in affected cattle herds (Dubey et al., 1992; Barr et al., 1993; Barr et al., 1994). *N. caninum* tachyzoites share fast proliferative properties with other parasite genera of the Sarcocystidae family with veterinary and human medicine relevance, such as *Toxoplasma*, *Besnoitia,* and *Sarcocystis*. However, unlike polyxenous *Toxoplasma gondii*, *N. caninum* is vastly host species-specific and its development is restricted to distinct host cell types *in vivo*, such as endothelial cells, neurons, epithelial cells, and fibroblasts (Hemphill et al., 1996; Lei et al., 2005). In general, apicomplexan parasites significantly modulate their host cells to sustain intracellular development and proliferation. Consequently, these parasites were described to alter numerous host cell functional categories, such as apoptosis (Goebel et al., 2001; Molestina et al., 2003; Lang et al., 2009), cytoskeleton (Dobrowolski and Sibley, 1996; Hermosilla et al., 2008), host cell membrane composition (Graewe et al., 2011), carbohydrate or cholesterol metabolism (Coppens et al., 2000; Nishikawa et al., 2005), innate immune reactions (Hermosilla et al., 2006; Taubert et al., 2006) and cell cycle progression (Brunet et al., 2008; Molestina et al., 2008; Velásquez et al., 2019; Velásquez et al., 2020a; Velásquez et al., 2020b). Likewise, it was demonstrated that *N. caninum* infections effectively alter host cellular immune responses by inducing distinct chemokine and adhesion molecule transcription in bovine endothelial cells (Taubert et al., 2006) or by triggering a pro-inflammatory gene transcription profile in bovine macrophages and trophoblasts (Jiménez-Pelayo et al., 2019; García-Sánchez et al., 2020). Additionally, global proteomic and transcriptomic analyses revealed a multitude of functional categories to be modulated by *N. caninum* in bovine trophoblasts, such as protein synthesis/turnover, metabolism, mitochondrial function, stress response, and host cell cycle (Horcajo et al., 2017; Regidor-Cerrillo et al., 2020). However, no data are currently available on either *Neospora hughesi* nor *N. caninum*-triggered alteration of this cellular function even though species-specific reactions are meanwhile well documented. Likewise, we recently reported that *T. gondii, B. besnoiti,* and *E. bovis* differentially affect cell cycle progression in the same host cell type [i. e. primary bovine umbilical vein endothelial cells (BUVEC)]. Thus, *T. gondii-*infected BUVEC showed G2/M-phase arrest, chromosome miss-segregation, and cytokinesis failure (Velásquez et al., 2019). In contrast, *E. bovis* and *B. besnoiti* infected BUVEC did not show chromosome segregation or cytokinesis impairment but failed to progress from G1-phase, suggesting a parasite-driven host cell cycle stasis in G1-phase or at G1-to-S-phase transition, possibly being related to specific requirements for their intracellular development (Velásquez et al., 2020a; Velásquez et al., 2020b).

Given that respective data are completely lacking on *N. caninum*-infected host cells, we here demonstrated the influence of *N. caninum* infection on host cell cycle progression and used the same primary host cell type (BUVEC) as before for *T. gondi-*, *E. bovis-* and *B. besnoiti-*related studies to generate comparative data. To the best of our knowledge, we here showed for the first time that *N. caninum* infections induce an S-phase arrest in host cell cycle progression, controlling not only the DNA amount but also some key proteins which are involved in the regulation of various cell cycle phases. As an interesting finding, we here documented that nuclei of *N. caninum-*infected BUVEC were reduced in size and showed altered morphologies over time. Moreover, the structure of their nuclear lamina changed, probably as a consequence of lamin B1 defects or due to actin-cytoskeleton disruptions (actin cap) occurring around the parasitophorous vacuole (PV) containing *N. caninum* tachyzoites.

## Results

### *N. caninum* Tachyzoite Development in Primary Bovine Umbilical Vein Endothelial Cells

**FIGURE 1:**
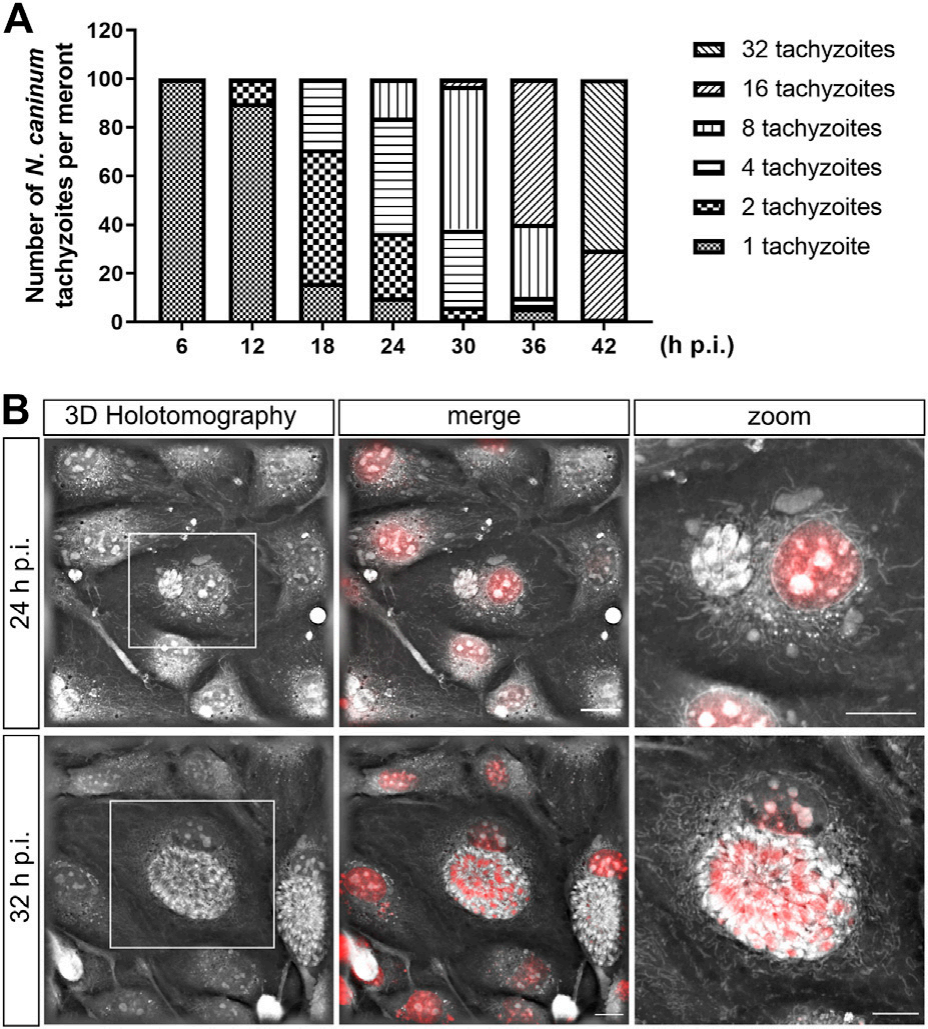
*N. caninum* tachyzoite intracellular development in primary bovine umbilical vein endothelial cells (BUVEC). Sub-confluent BUVEC were infected with *N. caninum* tachyzoites at an MOI 3:1 and analyzed from 6 to 42 h p. i. **(A)** Fixed cells were stained against *N. caninum* tachyzoite, and the tachyzoite numbers/parasitophorous vacuole (PV) were counted every 6 h during 42 h of infection. Mature tachyzoites were observed between 36 and 42 h p. i. The graph shows the distribution of the number of tachyzoites per meront at each time point. In BUVEC, the first duplication of *N. caninum* tachyzoites was observed only after 6 h p. i. Cells were lysed after 42 h p. i, thereby releasing newly formed tachyzoites into the medium. **(B)** Live cell 3D-holotomography images of *N. caninum* tachyzoite-infected BUVEC at 24 and 32 h p. i. nuclei of both, i. e., BUVEC and parasites, were stained with DRAQ5 (vital DNA staining, red). Bars: 5 μm.

Given that the developmental kinetics of apicomplexan parasites depend not only on the species but also on the host cell type, we here thoroughly monitored *N. caninum* tachyzoite formation over time in BUVEC. As expected, proliferation of *N. caninum* tachyzoites took slightly longer than that of *T. gondii* or *B. besnoiti* tachyzoites in the same host endothelial cell type (Velásquez et al., 2019; Velásquez et al., 2020a). To strictly avoid variations due to the seeding process, BUVEC isolate or tachyzoite batch, we here seeded identical BUVEC isolates at the same time point and used tachyzoites from the same isolation for host cell infections. By sticking to this procedure, we estimated tachyzoite numbers per meront throughout *in vitro* infection (i. e., at 6, 12, 18, 24, 30, 36, and 42 h p. i.). Analyses were restricted to 32 h p. i., since thereafter cell lysis was common. In each sample, a total of 100 host cells were analyzed. At the stage of 32 tachyzoites/PV, the meront was considered mature (Figure 1B). Overall, by applying an MOI of 1:1, we achieved a mean infection rate of 46.2 ± 3.4% in BUVEC. Developmental monitoring revealed that the first tachyzoite duplication took place between 6 and 12 h p. i., whilst the second and third steps of replication occurred between 12 and 18 h p. i. (Figure 1A). At 24 h p. i., most tachyzoites (90 ± 4.7%) had divided at least once. Consequently, PVs showed 2 (26.7 ± 1.4%), 4 (47.3 ± 1%), or 8 (16 ± 0.7%) tachyzoites (Figure 1A). At 30 h p. i., PVs mainly contained 8 (59 ± 0.3%) tachyzoites and, to less degree, 2 (1.33 ± 0.19%), 4 (31.2 ± 1.2%) or 16 (3 ± 0.3%) tachyzoites. At 36 and 42 h p. i., most PVs carried 16 (35.2 ± 0.4%) or 32 tachyzoites, respectively (Figure 1A). In order to monitor intracellular development of *N. caninum* under live cell conditions, we additionally performed live cell 3D-holotomographic microscopy covering up to 42 h of infection. Here, parasite development and nucleus formation were documented via vital DNA staining with the cell-permeable fluorescent DNA probe DRAQ5 (Figure 1B). Based on the formation of parasite rosettes over time and a concurrent lack of host cell lysis, we chose the time points of 24 and 32 h p. i. for further analyses on the impact of *N. caninum* replication on host cell cycle progression.

### *N. caninum* Infection Arrests the Host Cell Cycle in the S-Phase

**FIGURE 2:**
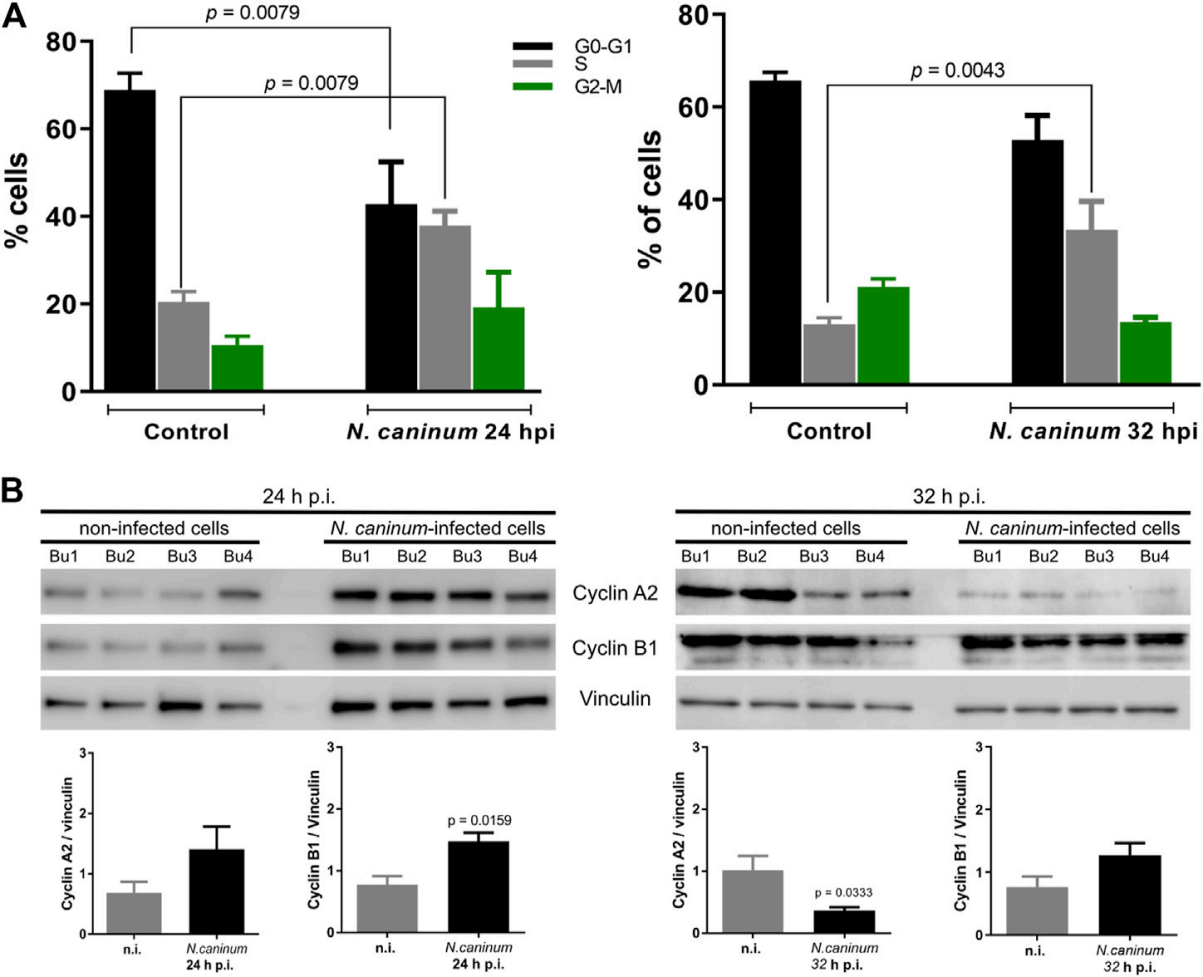
Effects of *N. caninum* infection on the host cellular cell cycle and on cyclin A2 and B1 abundance. Sub-confluent BUVEC (*n* = 6) were infected with tachyzoites of *N. caninum* at an MOI of 3:1 and examined for DNA contents at 24 and 32 h p. i. by FACS-based analyses. **(A)** Cell population was first gated in order to eliminate debris from the analysis. Furthemore, the DNA channel vs. the population histogram was used to obtain the total number of cells in each peak. Data were plotted as a percentage of total cells vs. DNA amounts found in each G0/G1-, S- and G2/M-phase, respectively. **(B)** Analysis of cell cycle-related molecule expression in *N. caninum*-infected BUVEC. Four biological replicates of BUVEC were infected with *N. caninum* tachyzoites and analyzed by Western blotting for the abundance of the cell cycle-related molecules cyclin A2 (S-, G2-phase transition), and cyclin B1 (M-phase), at 24 and 32 h p. i. The density of protein signals was quantified and graphed as a relative ratio to vinculin (loading control). Bars represent the median ±SD of four biological replicates.

In a first experimental approach, cell cycle progression in *N. caninum*-infected BUVEC was evaluated at 24 and 32 h p. i. by flow cytometry-based analysis of the cellular DNA content, allowing for the discrimination of the main three periods of the cell cycle: G0/G1-, S-, and G2/M-phase. This is a well-established method which proved suitable for other apicomplexan parasite-infected BUVEC (Velásquez et al., 2019; Velásquez et al., 2020a). An exemplary illustration for *N. caninum*-infected BUVEC gating is shown in Supplementary Figure S1). The data revealed a significantly increased proportion of infected host cells experiencing S-phase at both 24 and 32 h p. i. (infected vs. control cells: 24 h p. i.: *p* = 0.0079; 32 h p. i.: *p* = 0.0043) being accompanied by a simultaneous decrease of *N. caninum*-infected BUVEC in G0/G1-phase (24 h p. i.: *p* = 0.0079; 32 h p. i.: *p* = 0.049) when compared to non-infected control cells, thereby overall indicating a parasite-driven arrest of host cells in S-phase during full parasite proliferation (Figure 2A).

In a second experimental series, we measured the cellular abundance of selected key molecules of the cell cycle by analyzing four different *N. caninum*-infected and control BUVEC isolates via Western blotting to discriminate cell cycle phases in more detail (Figure 2B). Therefore, cyclin B1 and cyclin A2 abundance were monitored at both 24 and 32 h p. i. In line with current DNA content-based data, the abundance of cyclin A2, which is typically increased throughout S-phase peaking in G2-phase, was reduced in the M-phase at 32 h p. i., but was not altered at 24 h p. i. (*p* = 0.1229). Furthermore, cyclin B1, which starts to be upregulated in S-phase and peaks at the G2/M border, was found significantly enhanced in *N. caninum*-infected BUVEC at 24 h p. i. (*p* = 0.0159) but showed no significant changes in its abundance at 32 h p. i. when compared to control cells (*p* = 0.200). These cyclin-related data support results in Figure 2A about *N. caninum*-infected host cells failing to enter into the G2/M phase and arresting cells in the S-phase of the cell cycle.

### *N. caninum* Infection Alters S-Subphase Distribution in Host Endothelial Cells

**FIGURE 3:**
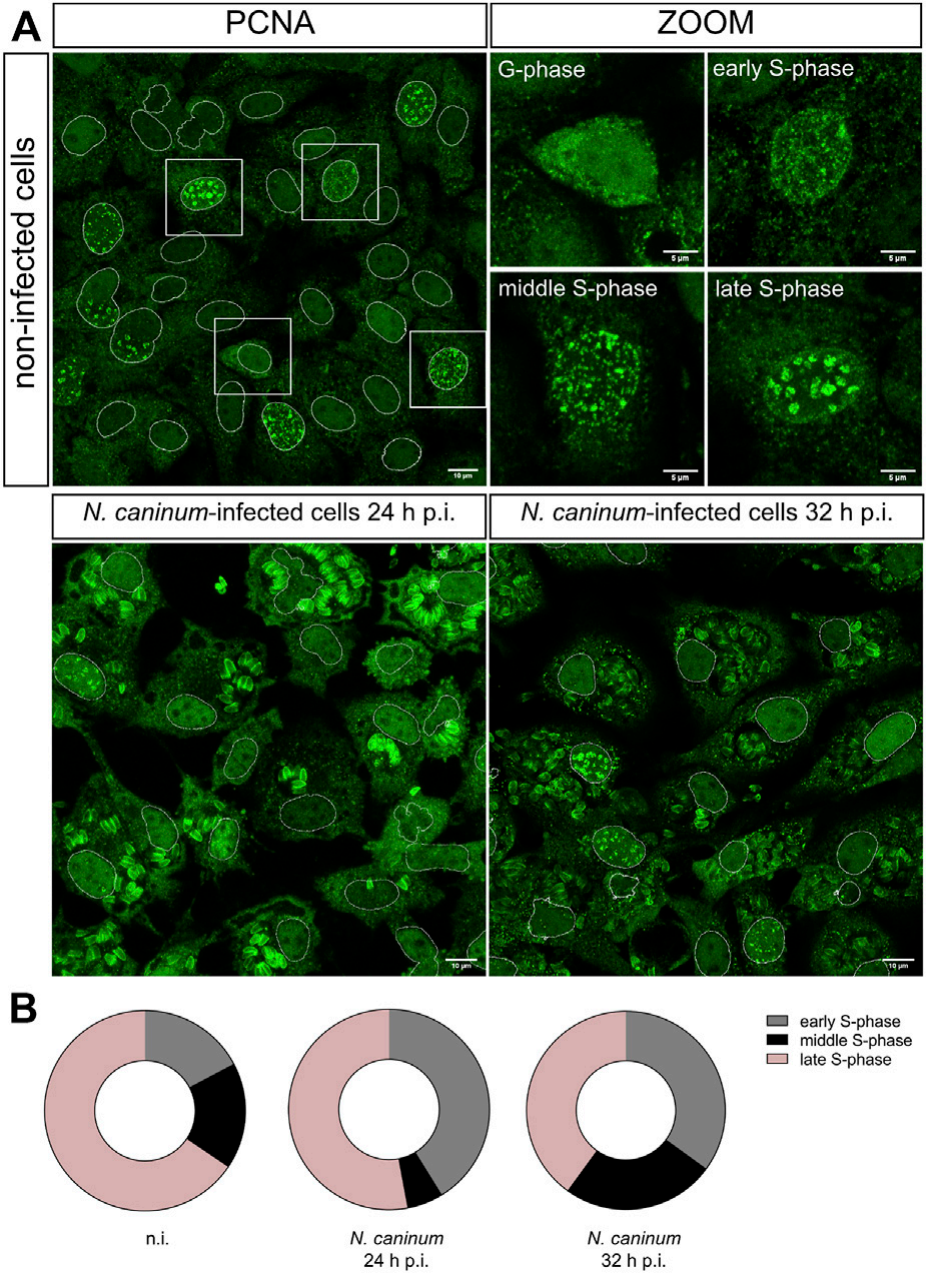
Effects of *N. caninum* infection on S-subphases. **(A)** *N. caninum*-infected BUVEC and non-infected cells were fixed at 24 ad 32 h p. i and stained for DAPI and proliferation cell nuclear antigen (PCNA; green) and analyzed *via* confocal microscopy. The nuclear PCNA localization and pattern allow one to discriminate the S-phase steps (early, middle, and late). The host cell nuclei were automatically segmented on the DAPI channel, the nuclear region was selected, and the coordinates were overlayed into the PCNA channel to identify the nuclear zone (white circles around the nuclei). **(B)** Quantification of nuclear PCNA-related pattern in non-infected cells in comparison with *N. caninum-*infected cells at 24 and 32 h p. i. The graph represents the percentages of cells in each S-phase step, early (gray), middle (black), and late (pink). The scale bar represents 5 µm.

Cell cycle progression is tightly controlled by different checkpoints regulating the entry into the subsequent phase or the exit from the current individual cell cycle phase. This regulation drives cells to either proceed to the next phase or to return to the last step. Irregularities occurring within each cell cycle phase may also block cell cycle progression. As shown above, *N. caninum-*infected BUVECs were arrested in S-phase, which may also result from irregularities during S-phase itself. Therefore, we monitored different subphases of S-phase by detecting PCNA protein, which is involved in DNA replication/repair and additionally has cell cycle-dependent properties. Of note, the characteristic nuclear PCNA distribution pattern is generally accepted as indicative of different S-subphases and therefore allows discrimination of early, mid, and late S-phase (Schönenberger et al., 2015). To analyze the nuclear PCNA pattern in *N. caninum*-infected BUVEC and controls, host endothelial cells were submitted to simultaneous PCNA immunodetection and nuclear staining (by DAPI) (Figure 3A). In non-infected BUVEC monolayers, most cells were in late S-phase (65.5 ± 9.1%), whilst fewer cells experienced early (17.2 ± 3.3%) or mid S-phase (17.2 ± 4.3%) (Figures 3A,B). However, considerable differences in S-subphases were detected in *N. caninum*-infected host cell layers. Thus, at 24 h p. i., a shift in S-subphases was apparent, thereby leading to an almost complete lack of host cells in mid S-phase (5.88 ± 2.4%) and a reduced proportion of BUVEC in late S-phase (52.94 ± 10.36%) but to an increase of host cells experiencing early S-phase (41.2 ± 8.7%) (Figure 3B). At 32 h p. i., infected host cells still showed a reduced proportion of host cells in the late S phase (40 ± 6.1%) when compared to control cells, whilst more host cells had again proceeded into the mid S-phase (25 ± 3.8%) (Figure 3B). Overall, it seemed that parasite-infected cells experienced difficulties in their progress into the late S-phase, which may then lead to an impairment in the transfer into the G2/M phase.

### *N. caninum* Infection Affects the Nuclear Structure and Perinuclear Actin Structures in Host Cell Layers

**FIGURE 4:**
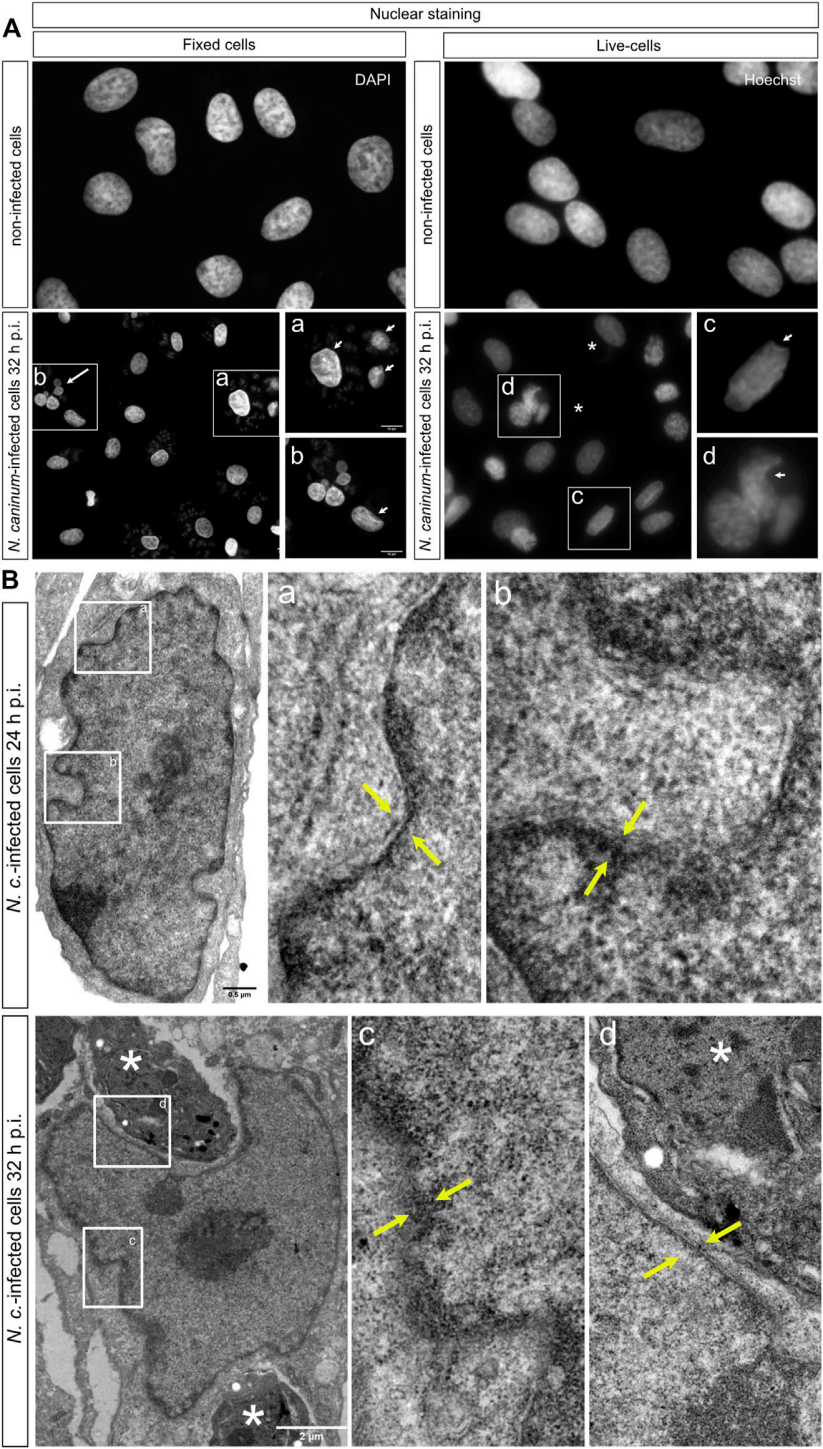
Morphology of cell nuclei in *N. caninum*-infected BUVEC layers. **(A)** Three BUVEC biological replicates were infected with *N. caninum* tachyzoites, and the host cell nuclear shape was compared between PFA-fixed and living cells after 32 h p. i. After fixation, cells were permeabilized and stained with a DAPI probe to allow host cell nucleus recognition as well as to detect the nucleus of the parasite (little gray dots surrounding the host cell nucleus). Live-cell imaging was developed with Hoechst nuclear staining, which allows a good resolution of the host cell nuclei but deficient parasitic nuclei, for that, the localization of the parasite is shown with an asterisk. **(B)** TEM analysis of *N. caninum-*infected host cells shows a nuclear compartment with depression and altered by tachyzoite localization (asterisk). The inspection of the nuclear membrane displayed zones without the detection of double membranes (space between yellow arrows). **(a, b)** Inset of the image into the left corner in order to show in more detail the nuclear membrane structure. The scale bar represents 5 µm.

When analyzing PCNA distribution within the nuclei of fixed *N. caninum*-infected BUVEC, we noticed irregular nuclear morphologies in both *N. caninum*-infected cells and non-infected cells within the same infected cell monolayer. Overall, the nuclear shape and volume play a central role in cellular and developmental processes and are maintained by mechanical forces mediated via cytoskeletal elements from inside and outside the cell. Typically, there is a correlation between cell shape and nuclear size, which is preserved during the entire cell life, and it is also inherited by daughter cells (Khatau et al., 2009). To verify the above mentioned morphological observations and to exclude fixation-based artefacts, we illustrated the nuclear morphology in both living and PFA-fixed cells via DNA staining with Hoechst 33342 and DAPI, respectively (Figure 4A). As expected, the nuclei of fixed cells from non-infected cell layers mainly showed a regular, oval shape with smooth cell borders, and a few cells displayed slight nuclear depressions (Figure 4A), the latter of which was practically absent in living (non-fixed), non-infected BUVEC monolayers (Figure 4A). In contrast, in PFA-fixed *N. caninum-*infected host cell layers, an increased proportion of cells showed nuclear abnormalities. These host cells no longer presented smooth borders and regular shapes of the nuclei but instead displayed irregular nuclear morphologies with inconsistencies, corners, strictures, and dentings (Figure 4A, white arrows), some of them unveiling nuclear fragmentation (Figures 4A,B, white arrow). In line, similar effects were observed in living cells, where nuclei showed invaginations and condensed lines within the nuclear area (Figures 4A–C, white arrow). Interestingly, we additionally observed several nuclei presenting a half-moon shape, which was not found in fixed cells (Figures 4A–D, white arrows). To confirm these morphological alterations, the nuclear structures were additionally illustrated by TEM analysis. In line with the above observations, *N. caninum-*infected host cells showed irregular, deformed nuclei at 24 and 32 h p. i. with invaginations and stretches of nuclear membrane disintegration (Figure 4B–space in between yellow arrows). Interestingly, we also observed a *N. caninum* tachyzoite being located within a nuclear invagination but seemingly lacking a PV (Figure 4B–asterisk 32 h p. i.).

**FIGURE 5:**
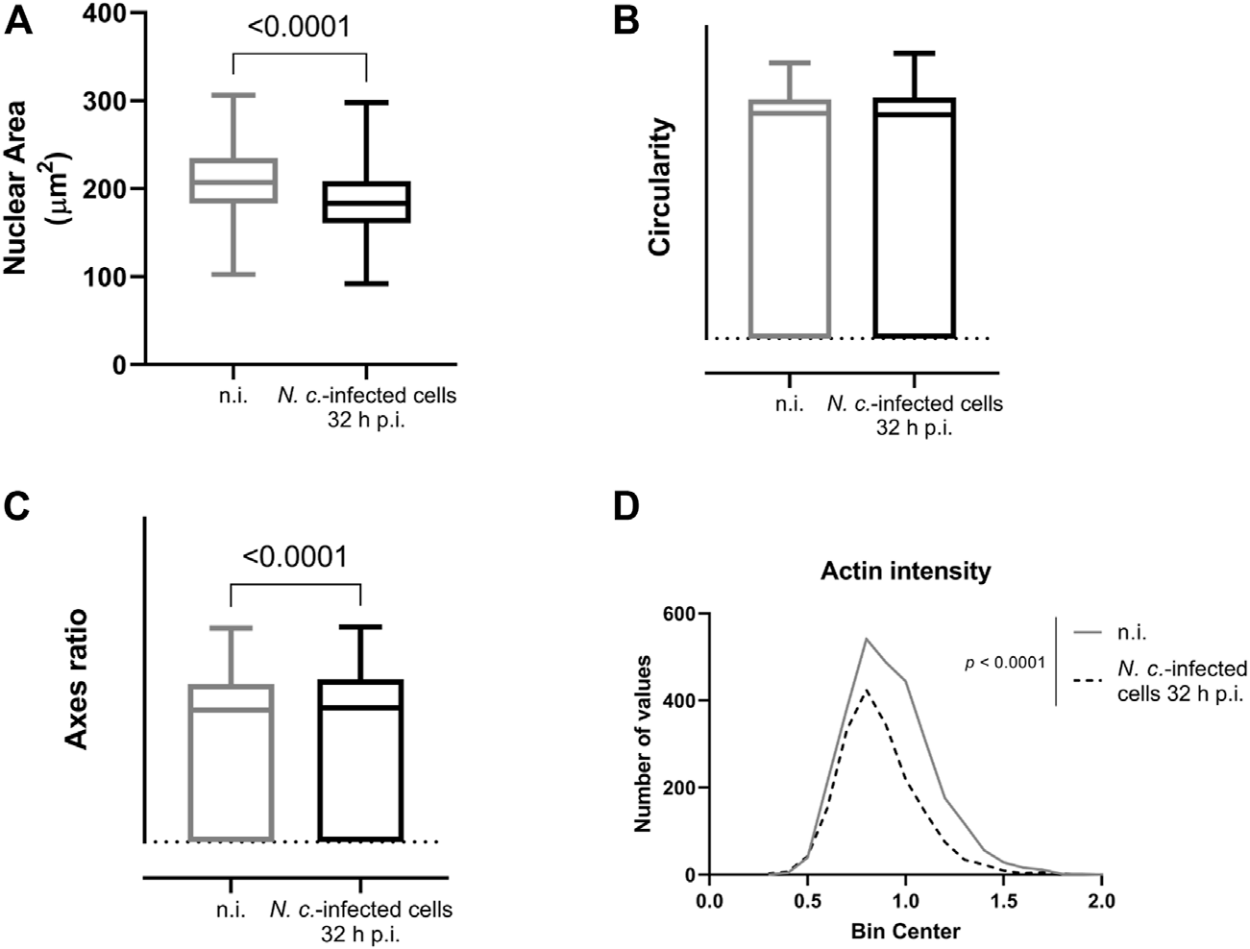
Effects of *N. caninum* infection on classical nuclear parameters. Non-infected and *N. caninum-*infected host endothelial cells (BUVEC) were stained with DAPI, and random pictures were taken at 20X for measuring the host cell nuclear area **(A)**, circularity **(B)**, nuclear axis ratio **(C)**, and the actin intensity **(D)**. Bars represent the median ±SD of three biological replicates.

To assess some general nuclear parameters and to confirm infection-driven nuclear changes on a quantitative level, we further measured the area, circularity, and nuclear axis ratio in both non-infected and infected cells. These analyses showed that the nuclear area was 11.7% smaller in *N. caninum-*infected host cells at 32 h p. i. when compared to control cells (Figure 5A), whilst the nuclear circularity was not affected by infection (Figure 5B). Furthermore, the axes ratio was revealed to be 4.9% larger for *N. caninum-*infected cells when compared to non-infected cells (Figure 5C).

**FIGURE 6:**
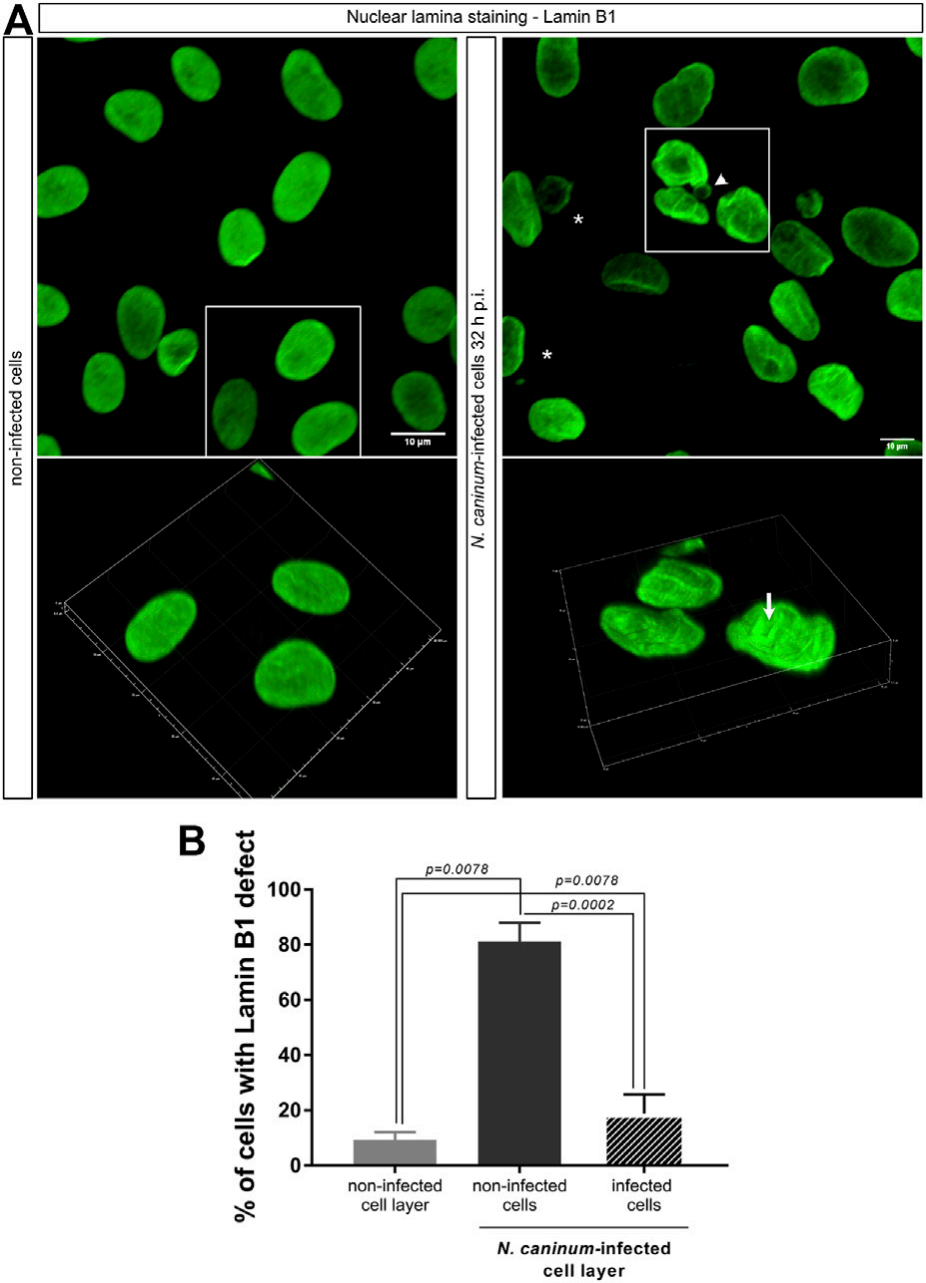
Nuclear lamin B1 detection in *N. caninum*-infected BUVEC layers. **(A)** Nuclear lamina analysis by the staining of lamin B1 (green). Fixed cells were stained against lamin B1 for the nuclear lamina and DAPI for the nuclear area recognition (not shown in the image). A white arrowhead in the 3D volume projection image: circular structures protruding from the nucleus; asterisks: parasite localization; white arrow: lamin B1 folding. **(B)** Quantification of the percentage of host cells with distorted lamin B1 localization in the total *N. caninum-*infected BUVEC monolayer (dark and light gray). The scale bar represents 10 µm. Bars represent the median ±SD of three biological replicates.

To study nuclear elements in more detail, we additionally analyzed the staining pattern of lamin B1 as a marker of the nuclear lamina. In general, the nuclear shape is maintained by several factors, which include both nuclear and cytoplasmic molecules. The nuclear envelope is formed by two concentric membranes being equipped with nuclear pore complexes and supported by an underlying lamina composed of a meshwork of nuclear intermediate filaments formed by A- and B-type lamins (Hetzer, 2010). Lamin B1 forms part of the nuclear lamina but also interacts directly with chromatin (Brunet et al., 2019). To analyze whether nuclear alterations were based on an irregular nuclear lamina formation, we analyzed the distribution pattern of lamin B1 in non-infected and *N. caninum-*infected (32 h p. i.) cell monolayers. As expected and in line with the above mentioned observations on DAPI-stained nuclei, non-infected cells showed a homogenous distribution of lamin B1 over the total nuclear area and only a very few cells eventually displayed tiny membrane foldings (Figure 6A). In contrast, a much higher proportion of host cells revealed altered nuclear lamin B1-related distribution patterns within *N. caninum*-infected BUVEC layers, which were characterized by inhomogeneous staining showing several nuclear foldings and invaginations, thereby reflecting abnormal nuclear shapes and irregular silhouettes (Figure 6A–white arrow in *N. caninum-*infected host cells Supplementary Video S1). In some nuclei, bubble-like protrusions (Figure 6A -white head arrow) were detected and further confirmed by 3D reconstructions (Supplementary Video S1). When assessing these nuclear abnormalities on a quantitative level, a significantly increased proportion of both *N. caninum-*infected (18.8 ± 6.9%) and non-infected (81.2 ± 6.9%) cells within an infected cell layer (infection rate: 25.96 ± 14.73%) showed inadequate lamin B1 distribution patterns and misshaped nuclei (Figure 6B) when compared to cells from non-infected cell layers (9.3 ± 2.8%) (infected/non-infected cells from infected layer vs. non-infected cell layer: both *p* = 0.0078).

**FIGURE 7:**
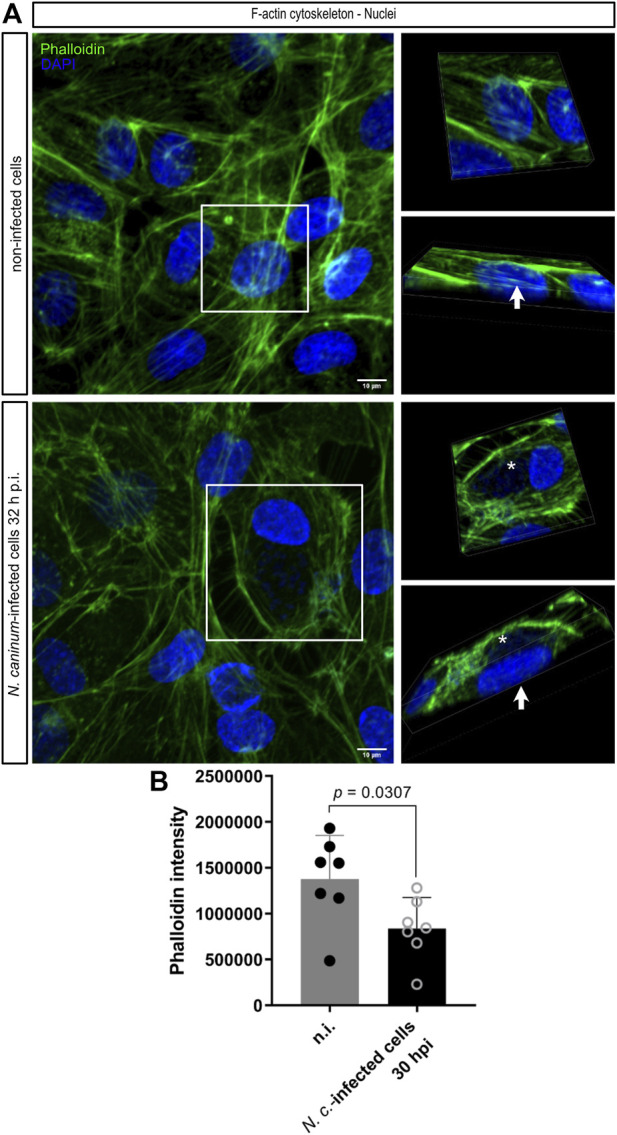
F-actin-based cytoskeletal nuclear structures in *N. caninum*-infected BUVECs. **(A)** Three biological BUVEC replicates were infected with *N. caninum* tachyzoites and, after 32 h p. i., submitted to F-actin (green) and DAPI (blue) staining. Pictures of the whole cell were taken in order to visualize the actin network in both cell conditions. In the right panel, the 3D volume image reconstruction of only one host cell is shown to display in detail the actin filament localization inside the cell. The white arrows show the nuclear region covered by the actin cytoskeleton, and the asterisks point out the parasite localization. **(B)** FACS-based quantification of the F- actin in both non-infected and *N. caninum-*infected host cells. Scale bar represents 10 µm. Bars represent the median ±SD of six biological replicates.

The shape of the total cell but also of its nucleus completely depends on the presence of distinct filamentous proteins, such as actin and intermediate filaments, as well as microtubules. Of special interest for nuclear shaping and function is the so-called perinuclear actin cap, which is composed of thick, parallel, and highly contractile actomyosin filament bundles with their ends being anchored in focal adhesions and the central part spanning over and being physically attached to the nucleus and nuclear lamina by the linker of nucleoskeleton and cytoskeleton (LINC) complex (Khatau et al., 2009; Kim et al., 2013; Maninova et al., 2017). To detect cytoskeletal actin filaments in non-infected and *N. caninum-*infected (32 h p. i.) host cells, we here used phalloidin staining (Figure 7A). When considering the total actin-based cytoskeletal network of cells, seemingly similar phenotypes were observed in both conditions (Figure 7A). However, the area in juxtaposition to nuclei appeared considerably affected in *N. caninum*-infected cells. Thus, actin filaments typically transversing the nuclei and being anchored to the nuclear periphery–as commonly observed in non-infected cells (Figure 7A–indicated by an arrow, Supplementary Video S2) - were consistently absent from the nuclei of *N. caninum-*infected cells (Figure 7A–indicated by asterisk). Consequently, the remnants of the respective filaments seemed fragmented or eventually without anchorage. Interestingly, actin quantification by FACS showed decreased abundance in *N. caninum*-infected cells (*p* = 0.0240), thereby mirroring the observation that perinuclear actin intensities seemed reduced in *N. caninum*-infected cells when compared to control cells. In contrast to lamin B1-related data, the actin cap-based changes were not exclusively found in infected cells, thereby questioning this finding as an exclusive mechanistic basis of nuclear malshaping. However, these overall observations suggest that *N. caninum* intracellular development might interfere with the host cellular actin cytoskeleton in the perinuclear region and most likely lead to nuclear membrane destabilization, thereby contributing to abnormal nuclear shaping.

**FIGURE 8:**
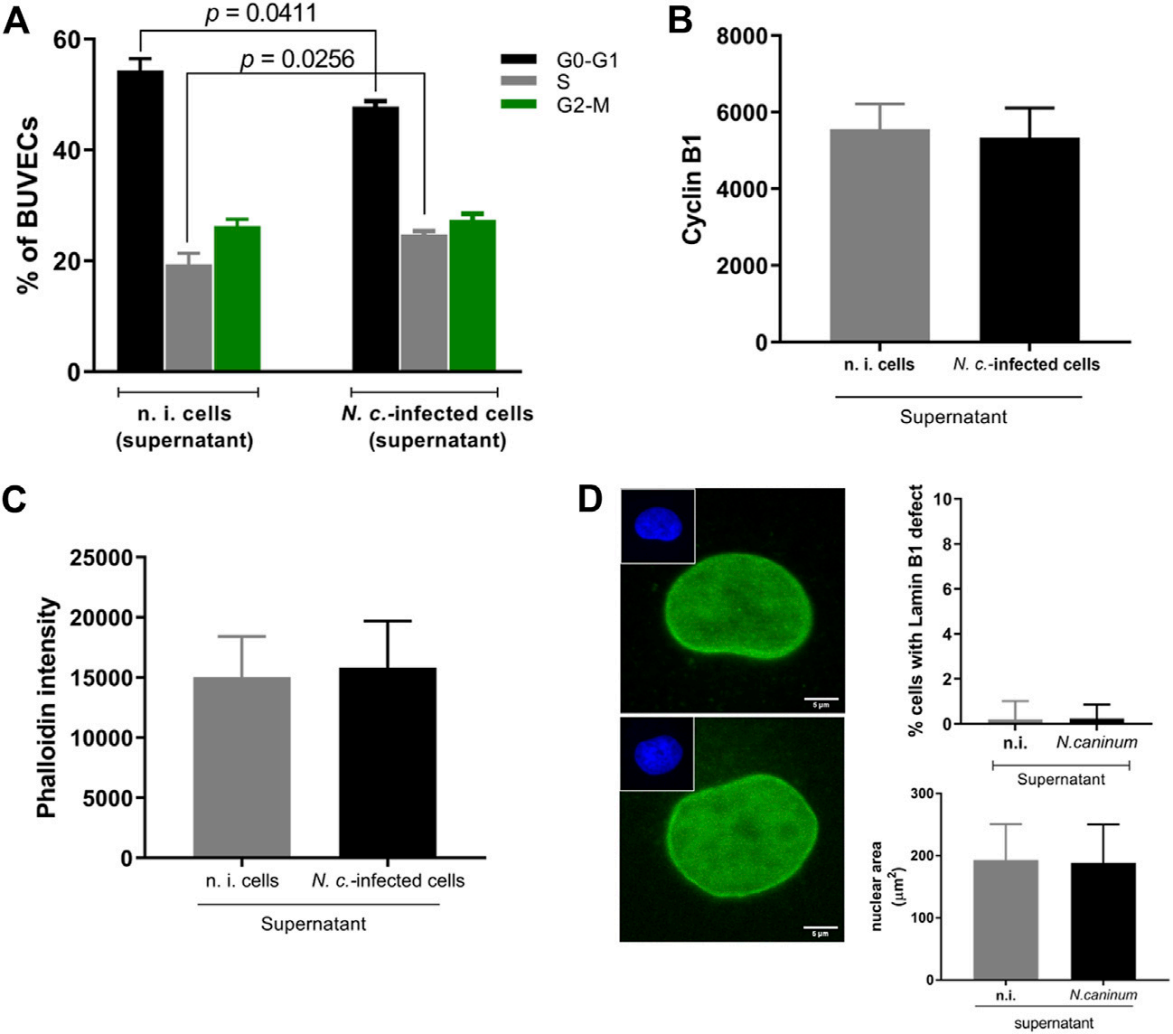
Analysis of *N. caninum* infection-derived supernatant paracrine effects on host cell cycle progression and the nuclear shape of non-infected host cells. Six biological BUVEC (*n =* 6) replicates were infected with *N. caninum* tachyzoites and, after 32 h p. i., the medium (i. e., supernatant) was isolated and filtered to erase any contamination with tachyzoites/cell debris. The non-infected and *N. caninum-*infected host cell supernatant was immediately transferred to non-infected monolayers and incubated for 32 h in order to analyze paracrine effects on host cell cycle progression and nuclear shape. **(A)** BUVEC populations were first gated, and the histograms for propidium iodide (PI) were conducted in order to differentiate the mean peaks of the histograms. Data were plotted as the percentage of total cells vs. DNA amounts found in each G0/G1-, S- and G2/M-phase. **(B, C)** Analyses of cyclin B1 and F-actin by FACS-based quantification. **(D)** Lamin B1 detection by immunofluorescence (green) and the nuclear area by DAPI (left inset) staining. The nuclear size was quantified using the DAPI channel and represented in the graph. Bars represent the median ±SD of six biological replicates. The scale bar represents 5 µm.

Nuclear lamin B1-related data showed that non-infected host cells within infected monolayers revealed a higher percentage with abnormal lamin B1 distribution in comparison to *N. caninum*-infected ones. To test for *N. caninum*-driven paracrine effects on nuclear lamin B1 in non-infected cells, we tested whether supernatants from *N. caninum*-infected BUVEC monolayers would lead to nuclear lamin B1 abnormalities in non-infected BUVEC layers. Therefore, non-infected BUVEC were supplemented with infection-conditioned medium (i. e. filtered supernatants of *N. caninum* tachyzoite-infected BUVEC after 32 h p. i.) or with supernatants from non-infected BUVEC (controls). The results demonstrated that supernatants from *N. caninum*-infected BUVEC indeed induced S-phase arrest (*p* = 0.0256) with a concomitant G0-G1 phase reduction (Figure 8A) in non-infected BUVEC. However, cyclin B1 abundance was not significantly influenced by differential supernatant treatments (Figure 8B). Moreover, phalloidin-based actin quantification showed no effect of differential supernatant treatments on actin-mediated nuclear shapes. Thus, neither actin-derived effects on nuclear shapes nor on nuclear sizes were observed in supernatant-treated cell monolayers (Figures 8C,D).

## Discussion

The cell cycle is a well-conserved and tightly regulated process in all eukaryotic cells and is key for cell survival/division. Up to date, studies on apicomplexa-driven cell cycle modulation have unveiled changes in abundance of cyclins critically involved in host cell division, such as G2-specific cyclin B1 or cyclins A/B in combination with cyclin E in case of *T. gondii* (Molestina et al., 2008; Velásquez et al., 2019), cyclin E1 in case of *B. besnoiti-* and *E. bovis*-infected host cells thereby indicating a cell cycle arrest at G1/S transition phase (Velásquez et al., 2020a; Velásquez et al., 2020b). Moreover, apicomplexa-modulated cell cycle progress seems to be parasite species- and cell type-dependent involving up- or downregulation of specific cyclins while performing this influence (Molestina et al., 2008; Taubert et al., 2010; Velásquez et al., 2019; Velásquez et al., 2020a; Velásquez et al., 2020b). Thus, *T. gondii* tachyzoites blocked host cell cycle progression in G2/M-phase, thereby interfering with chromosome segregation and mitotic spindle formation and inducing cytokinesis failure in infected endothelial cells (Velásquez et al., 2019). In contrast, infections of the same host cell type with *B. besnoiti* tachyzoites and *E. bovis* sporozoites led to host cell arrest in G0/G1-phase but altered the abundance of cell cycle-related key molecules in a different manner (Velásquez et al., 2020a; Velásquez et al., 2020b). As such, exclusive *E. bovis* infection triggered premature senescence in BUVEC, in addition to cyclin E1 up-regulation during macromeront formation (Velásquez et al., 2020b). Conversely, and despite the fact that *N. caninum* tachyzoites replicate in bovine endothelial cells of blood and lymph vessels *in vivo*, no data were available on *N. caninum-*mediated cell cycle modulation in host endothelial cells. Therefore, in the current study, we used BUVEC as the host cell system for the following reasons: *1*) to be as close as possible to the *in vivo* situation in bovines, and *2*) to avoid cell type-driven effects and thereby simplify reliable comparisons on parasite species levels when referring to other studies above mentioned. Here, we showed that *N. caninum* tachyzoites indeed interfered with host cell cycle progression by inducing S-phase arrest, nuclear lamin B1 maldistribution, peri-nuclear actin losses, and host cellular nuclear deformations. More importantly, these findings clearly differed from those alterations driven by different stages of the closely related parasites *T. gondii, B. besnoiti,* and *E. bovis*, indicating that apicomplexa-driven cell cycle modulation is not only a species- and cell type- but also a stage-dependent process*.*

By the combination of WB- and FACS-based analyses, we here demonstrated that *N. caninum-*infected host endothelial cells were arrested at the DNA synthesis stage when harboring immature meronts with no more than 8 tachyzoites onwards. One plausible explanation for cell cycle arrest in S-phase is that during this phase not only protein-, amino sugar- and pentose phosphate-synthesis are up-regulated but also metabolic pathways, such as glycolysis/gluconeogenesis and nucleotide sugar metabolism, all of them fundamental for both, host cell maintenance and intracellular parasite proliferation (Regidor-Cerrillo et al., 2020; Velásquez et al., 2021; Vélez et al., 2021). Given that tachyzoite production is an energy- and building block-demanding process, the transient or continuous deprivation of essential molecules could indeed induce an energy-lacking status in *N. caninum*-infected host cells, finally leading to a block in DNA synthesis and/or mitotic process. Likewise, recent data showed that *N. caninum* tachyzoites recruit host cell structures to their PV and salvage lipids from organelles (Nolan et al., 2015). Interestingly, treatments of non-infected endothelial cells with infection-conditioned medium also resulted in S-phase stasis, suggesting that either the parasites or *N. caninum-*infected endothelial host cells release soluble factors which act on bystander cells. Likewise, induction of S-phase (based on BrdU incorporation) by infection-conditioned medium was also described for *T. gondii*-driven effects in non-infected human foreskin fibroblasts (HFF) (Lavine and Arrizabalaga, 2009). Interestingly, besides inducing S-phase stasis in host cells, *T. gondii* more readily infected fibroblasts in S-phase and conditioned medium increased the efficiency of invasion in HFF, which led to the assumption that cells in S-phase bear a selective advantage for *T. gondii* (Lavine and Arrizabalaga, 2009).

Important molecular mechanisms involved in adequate cell cycle progression include the accuracy of DNA synthesis and several checkpoint-based controls. The later exist for G1-phase (G1-checkpoint), during the whole S-phase (S-phase checkpoint) and after S-phase (G2/M checkpoint) thereby covering a temporal and spatial program (Hartwell and Weinert, 1989; Kastan and Bartek, 2004). The current data suggests that *N. caninum* infection could lead to S-phase stasis of host cells by affecting the synthesis of proteins linked to distinct cell cycle checkpoints. It is well accepted that cyclin A2 is a key regulator of both mitotic entry and DNA replication, following a coordinated pattern of expression and subcellular localization (Pagano et al., 1992; Petersen et al., 1999; Fung et al., 2007). As such, the complex of cyclin A/cdk2 drives eukaryotic cells in G2-phase to continue into the mitosis phase. Early cyclin A/cdk2 downregulation correlates with S-phase arrest, not allowing cells to enter into the G2/mitosis phase (Furuno et al., 1999). Interestingly, our data showed that *N. caninum-*infected endothelial host cells were stuck in S-phase with a concomitant down-regulation of cyclin A2, suggesting that *N. caninum-*infected host cells were most probably not able to pass the cell cycle checkpoints after S-phase.

Unexpectedly, we observed that *N. caninum* drastically affected the nuclei of infected host cells, i. e., their sizes, the actin cap and the nuclear lamina. Of note, these nuclear features were not exclusively observed in *N. caninum*-infected host cells but also in non-infected bystander cells. The nuclear lamina is formed by lamins A and B, with lamin B being essentially involved in cell viability, DNA replication, DNA repair, RNA polymerase transcription, and chromatin remodeling as epigenetic control. Thus, the specification of distinct lamins and their localization affect the DNA elongation chain, being therefore considered fundamental for DNA synthesis during cell replication (Moir et al., 2000; Spann et al., 2002; Shimi et al., 2008; Shumaker et al., 2008). The current data on lamin B1 and nuclear shape revealed that *N. caninum*-infected host cells showed nuclei that were diminished in size and presented altered lamin B1 distribution, a phenomenon that may also be linked to their inability to overpass S-phase since nuclear lamina defects may hamper accurate DNA repair or DNA replication. As a consequence, critical DNA-related damage checkpoints failed and cells were not able to progress into the mitotic process. Small nuclei originating from lamin-depleted nuclear extracts showed an inhibition of nuclear transport and thereby resulted in cell cycle arrest prior to DNA synthesis (Newport et al., 1990; Meier et al., 1991). Besides lamin B1-related nuclear alterations, we additionally observed actin-related abnormalities in *N. caninum*-infected BUVEC. Thus, the current data indicated that the juxta- and transnuclear actin cytoskeleton was not properly formed and that especially the perinuclear actin cap was lacking in infected cells. Importantly, perinuclear actin cap integrity depends on proper F-actin assembly, which is critically involved in adequate nuclear shaping (Khatau et al., 2009). Given that no F-actin-mediated nuclear shape alterations were detected in non-infected host cells exposed to infection-conditioned medium. Moreover, we suggest that the reduction of host cell nucleus sizes as well as the cell cycle arrest in S-phase could be a consequence of DNA damage caused by fast intracellular meront growth and resulting in isometric shrinkage of host cell nuclei.

Taken together, the current and recent data underline species and even stage-specific effects on host cell cycle progression that our group has published. This suggests that further investigations into parasite species of the family Sarcocystidae are needed to elucidate precise molecular mechanisms, signaling pathways, and cyclins involved in individual modulation of cell cycle progression. In summary, we here demonstrate to our best knowledge for the first time that *N. caninum* tachyzoite infection affects host endothelial cell cycle progression in a species-specific manner by alterations in cyclin A2/B1 abundance and actin-dependent nuclear lamina deformations, resulting in irregularities in S-phase and S-to-G2/M-phase transition.

## Materials and Methods

### Primary Bovine Umbilical Vein Endothelial Cell Isolation and Maintenance

BUVEC were isolated from umbilical veins obtained from calves born by *sectio caesarea* at Justus Liebig University Giessen, Giessen, Germany. Therefore, umbilical cords were kept at 4°C in 0.9% HBSS-HEPES buffer (pH 7.4; Gibco, Grand Island, NY, United States) supplemented with 1% penicillin (500 U/mL; Sigma, St. Louis, MO, United States) and streptomycin (500 μg/ml; Sigma) for a maximum of 16 h before use. For endothelial cell isolation, 0.025% collagenase type II (Worthington Biochemical Corporation) in Pucks solution (Gibco) was infused into the lumen of ligated umbilical veins and incubated for 20 min at 37°C in a 5% CO_2_ atmosphere. After gently massaging the umbilical veins, the cell suspension was collected and supplemented with 1 ml of fetal calf serum (FCS, Gibco) to inactivate collagenase. After two washes (350 x *g*, 12 min, 20°C), cells were suspended in complete endothelial cell growth medium (ECGM, PromoCell, supplemented with 10% FCS), plated in 25 cm^2^ tissue culture flasks (Greiner) and kept at 37°C in a 5% CO_2_ atmosphere. BUVEC were cultured in modified ECGM medium [EGCM, diluted at 30% in M199 medium (Greiner), supplemented with 5% FCS (Greiner), 1% penicillin and streptomycin (both Greiner)] with medium changes every 2–3 days. BUVEC cell layers were used for infection after 3 passages *in vitro*. All BUVEC isolations were conducted by the Institutional Ethics Commission of Justus Liebig University of Giessen (Germany) and by the current European Animal Welfare Legislation: ART13TFEU.

### Parasite Maintenance

*N. caninum* (strain Nc1) tachyzoites were maintained by serial passages either in BUVEC or African green monkey kidney epithelial cells (MARC-145) according to Taubert et al. (2006). Cell supernatants containing egressed parasites were collected and centrifuged once at 200 x *g* for 2 min to eliminate cellular debris. The supernatant was transferred to a new tube and centrifuged again at 800 x *g* for 6 min. The pellet was resuspended in modified ECGM medium and tachyzoites were then counted in a Neubauer hemocyte chamber.

Depending on the experiment, BUVEC (4-6 biological replicates) were either seeded in µ-dishes of 35 mm diameter (IBIDI, Martinsried, Germany) for live cell 3D-holotomographic microscopy (3D Explorer, Nanolive), in 25 cm^2^ culture flasks (Greiner) for FACS-based assays, or in 12-well plates (Greiner) containing fibronectin-coated (2.5 μg/ml, Thermo Fisher) glass coverslips (Nunc) for immunofluorescence assays. In all cases, plates were incubated at 37°C with a 5% CO_2_ atmosphere until confluency. Then, host endothelial cells were infected with 2.4 × 10^6^ (µ-dishes of 35 mm diameter), 2.4 ×10^5^ (12-well plates) or 8.4 × 10^6^ (25 cm^2^ culture flasks) fresh tachyzoites. Cell culture medium was changed 1 day after infection and thereafter every second day. Infection rates were determined at 1 day post infection (p. i.) microscopically.

### Monitoring of Intracellular *N. caninum* Tachyzoite Development in Bovine Umbilical Vein Endothelial Cells

Three BUVEC isolates were simultaneously seeded into 12-well plates containing 15 mm diameter fibronectin-coated (2.5 μg/ml, ThermoFisher) coverslips (Nunc). Three technical replicates were generated for each time point. All wells were infected at the same time. Samples were collected at different time points after infection (6, 12, 18, 24, 30, 36, and 42 h p. i.) and fixed in 4% paraformaldehyde (15 min, Roth). Tachyzoite development was analyzed by immunofluorescence using 4′,6-diamidin-2-phenylindol (DAPI) staining as a nuclear marker and an in-house canine hyperimmune serum to detect *N. caninum* stages. Randomly, the number of tachyzoites/PV were counted in ≥100 host cells per time point, BUVEC isolate and replicate.

### Live-Cell 3D-Holotomographic Microscopy Analysis

Refractive index (RI)-based 3D-holotomographic images were obtained by a live cell 3D Cell Explorer-Fluo microscope (Nanolive) equipped with a ×60 magnification (*λ* = 520 nm, sample exposure 0.2 mW/mm2) and a depth of field of 30 µm. Cells were seeded on µ-dishes (35 mm diameter, IBIDI, Martinsried, Germany) and infected with *N. caninum* tachyzoites (MOI: 1:2). Non-infected and *N. caninum*-infected BUVEC were transferred to a top-stage incubator (IBIDI, Martinsried, Germany) to control temperature, humidity, and CO_2_ levels during microscopy. Images were captured and analyzed using STEVE^®^ software (Nanolive) to obtain a RI-based z-stack. For nuclei detection, samples were stained with DRAQ5 Fluorescent Probe^®^ (5 μM, Thermo Fischer). Morphological alterations of host cell nuclei was evaluated by live cell imaging in non-infected and *N. caninum-*infected BUVEC stained with Hoechst 33,342 (0.2 μg/ml, ThermoFisher).

### Protein Extraction

Proteins were extracted from infected and non-infected BUVEC by cell sonication (20 s, 5 times) in RIPA buffer (50 mM Tris-HCl, pH 7.4; 1% NP-40; 0.5% Na-deoxycholate; 0.1% SDS; 150 mM NaCl; 2 mM EDTA; 50 mM NaF, all Roth) supplemented with a protease inhibitor cocktail (1:200, Sigma-Aldrich). Cell homogenates were centrifuged (10.000 *× g*, 10 min, 4°C) to sediment intact cells and nuclei. The RIPA buffer-soluble protein content of the respective supernatants was quantified via the Coomassie Plus (Bradford) Assay Kit (Thermo Scientific) following the manufacturer’s instructions.

### Sodium Dodecyl-Sulfate Polyacrylamide Gel Electrophoresis and Immunoblotting

For immunoblotting, protein samples were supplemented with 6 M urea protein loading buffer. After boiling (95°C) for 5 min, proteins (60 µg/slot) were separated in 12% or 15% polyacrylamide gels via electrophoresis (100 V, 1.5 h; tetra system, Bio-Rad). Proteins were then transferred to polyvinylidene difluoride (PVDF) membranes (Millipore) (300 mA, 2 h at 4°C). Samples were blocked in 3% BSA in TBS [50 mM Tris-Cl, pH 7.6; 150 mM NaCl containing 0.1% Tween (blocking solution); Sigma-Aldrich] for 1 h at room temperature (RT) and then incubated in primary antibodies (Table 1) diluted in blocking solution (overnight, 4°C). Detection of vinculin was used as a loading control for sample normalization. Following three washes in TBS-Tween 0.1% buffer, blots were incubated with secondary antibody (Table 1) solutions (dilution in blocking solution, 30 min, RT). Following three further washes in TBS-Tween (0.1%) buffer, signal detection was accomplished by an enhanced chemiluminescence detection system (ECL^®^ plus kit, GE Healthcare) and recorded using a ChemoCam Imager (Intas Science Imaging). Protein masses were controlled by a protein ladder (PageRuler Plus^®^ Prestained Protein Ladder ∼10–250 kDa, Thermo Fisher Scientific). Protein band intensities were quantified by the Fiji Gel Analyzer plugin (Schindelin et al., 2012).

**TABLE 1:** Primary and secondary antibodies used in the current study.

Antigen	Company	Cat. number	Origin/reactivity	Dilution
**Primary antibodies**				
Vinculin	Santa Cruz	sc-73614	mouse	1:1000
Cyclin A2	Abcam	ab38	mouse	1:1000
Cyclin B1	Abcam	ab32053	rabbit	1:3000
PCNA	Abcam	ab18197	rabbit	1:1000
Lamin B1	Abcam	ab16048	rabbit	1:2000
*Neospora caninum*	in-house	—	dog	1:50
**Secondary antibodies**				
Antigen/conjugate	Company	Cat. number	Host/target	Dilution
Goat anti-mouse IgG peroxidase-conjugated	Pierce	31430	goat/mouse	1:40,000
Goat anti-rabbit IgG peroxidase-conjugated	Pierce	31460	goat/rabbit	1:40,000
Alexa Fluor 594	ThermoFisher	R37117	goat/rabbit	1:500
Alexa Fluor 594	Jackson Immuno	304–585-003	rabbit/dog	1:500

### Immunofluorescence Assays

BUVEC layers were fixed with paraformaldehyde (4%, 15 min, RT; Roth), washed thrice with PBS and incubated in blocking/permeabilization solution (PBS with 3% BSA, 0.1% Triton X-100; 1 h, RT). Thereafter, samples were incubated in primary antibodies (Table 1) diluted in blocking/permeabilization solution (overnight, 4°C, in a humidified chamber). After three washes in PBS, samples were incubated in secondary antibody solutions (Table 1; 30 min at RT and complete darkness). Cell nuclei were labeled with DAPI-supplemented mounting medium (Fluoromount G, ThermoFisher).

### Detection of *N. caninum* Infection-Driven Paracrine Effects

To study potential *N. caninum* infection-driven paracrine effects on bystander cells, we incubated non-infected BUVEC isolates (*n* = 6) with filtered (0.2 μm filter) supernatants either originating from non-infected control BUVEC or from the same BUVEC isolates that had been infected with *N. caninum* for 32 h. After 24 h of supplementation, host cells were collected, fixed and analyzed by FACS reading for cell cycle phases (FxCycle Far^®^ red staining; Invitrogen) or probed for lamin B1 and actin.

### Flow Cytometry-Based Analysis of Cell Cycle Phases

Cellular DNA content was measured using the FxCycle Far red stain reagent (Invitrogen, F10348) according to the manufacturer’s instructions. The samples were analyzed by a BD LSRFortessa™ cell analyzer (Becton-Dickinson, Heidelberg, Germany) applying 633/5 nm excitation and emission collected in a 660/20 band-pass. Cells were gated according to their size and granularity. Exclusively morphologically intact host cells were included in the analysis.

### Image Acquisition and Image Reconstruction

Fluorescence images were acquired with a ReScan Confocal microscope instrumentation (RCM 1.1 Visible, Confocal. nl) equipped with a fixed 50 µm pinhole size and combined with a Nikon Ti2-A inverted microscope. The microscope was equipped with a motorized Z-stage (DI1500, Nikon). The RCM unit was connected to the Toptica CLE laser with the following excitations: 405/488/561/640 nm. Images were taken via an sCMOS camera (PCO edge) using a CFI Plan Apochromat ×60 lambda-immersion oil objective (NA 1.4/0.13; Nikon). The setup was operated by the NIS-Elements software (version 5.11). Images were acquired via z-stack optical series with a step size of 0.1 microns depth to cover all structures of interest within the analyzed host cells. The Z-series were displayed as maximum z-projections. Identical brightness and contrast conditions were applied for each data set within one experiment using Fiji software (Schindelin et al., 2012).

Further, images were edited by deconvolution software (3D deconvolution module, NIS-Element module, Nikon). The algorithm used for image deconvolution was selected depending on the structures to be shown in the pictures: Landweber for lamin B1 (20 iterations), Richardson-Lucy for phalloidin (20 iterations). Deconvoluted images were displayed as maximum z-projections, brightness, and contrast were adjusted using Fiji software (Schindelin et al., 2012). Deconvoluted z-stacks were submitted to the NIS-Element software volume viewer module, applying maximum intensity to z-projections.

Proliferation cell nuclear antigen (PCNA) localization analyses were performed by an automated selection of the nuclear area using the Fiji software, applying the following workflow: An Otsu threshold was subjected to the DAPI channel to obtain the total nuclear area. Particles larger than 800 pixels were selected and merged with the PCNA channel (nuclear selection is exemplary illustrated in Figure 3 as white circles surrounding the nuclei). The number of host cells in each S-subphase was counted manually according to the instructions given by Schönenberger et al. (2015). For nuclear size analysis, ROIs were measured using Fiji measure plugins following nuclear selection. Cell nuclei were segmented using Otsu thresholding as the binary image. Finally, morphological features (circularity, axes ratio, area and average intensity) were obtained using particle analysis in Fiji software. Actin-related average intensity per host cell was calculated using the pixel area enclosed in the nuclear masks.

### Transmission Electron Microscopy Analysis

Three BUVEC isolates were grown until confluence in T-75 cm^2^ culture flasks (Greiner), infected with freshly isolated *N. caninum* tachyzoites and cultured until 32 h p. i. Then cells were washed with PBS and fixed with 3 ml of fixing solution per flask (0.25% glutaraldehyde, 0.1 M cacodylate buffer and 4% PFA; Merck). After 2 min of treatment, the cells were gently scraped in larger pieces from the flask bottom to preserve the monolayer by using a rubberpolice (Greiner). After 24 h of fixation, samples were washed with 0.15 M Hepes buffer and stabilized with 1% osmic acid for 2 h. For contrasting, the samples were incubated overnight in half-saturated uranyl acetate solution (both Merck) and then washed with distilled water. Samples were dehydrated in an ascending ethanol series and finally embedded in Agar 100 resin (Agar scientific Ltd. United Kingdom). Ultrathin sections were cut using an ultramicrotome (Reichert Ultracut E, Leica) and examined in a transmission electron microscope (Zeiss EM 902). Digital images were captured with a slow-scan 2 K CCD camera (TRS, Tröndle, Moorenweis, Germany).

## Statistical Analysis

All data were expressed as mean ± SD from three independent experiments. In all cases, an unpaired *t*-test (non-parametric) was performed to compare infected and non-infected/non-treated data sets. Significance was defined as *p* ≤ 0.05. All graphs and statistical analyses were performed using GraphPad Prism^®^9 software.

## Data Availability

The original contributions presented in the study are included in the article/Supplementary Material; further inquiries can be directed to the corresponding author.

## Supplementary Material

Supplementary Figure S1 FACS-based analysis showing the gating strategy of BUVEC cells and the representative histogram. In a first step, the total cell population was gated according to shape and granularity of the cells **(A)**. In a second step, the PI-positive population was selected **(B)** and the representative histogram displaying main peaks for the G0/1 (2N)-, S- and the G2/M-phase (4N) was generated **(C)**.

Supplementary Video S1 Lamin B1 detection in *N. caninum*-infected cell layers (32 h p. i., 3D-reconstruction). PFA-fixed *N. caninum*-infected BUVEC were stained for Lamin B1 (green; the image corresponds to the one shown in Figure 7A). 3D reconstruction was performed by NIS element software built-in plugins. Background and signal levels were equally adjusted to illustrate nuclear membrane folding of the cells.

Supplementary Video S2 F- actin detection in *N. caninum*-infected cells (32 h p. i., 3D-reconstruction). PFA-fixed *N. caninum*-infected BUVEC were stained for F-actin (Phalloidin: green; the image corresponds to the one shown in Figure 7A). 3D reconstruction was performed by NIS element software built-in plugins. Background and signal levels were equally adjusted to illustrate the actin cytoskeleton in both non-infected and *N. caninum*-infected cells.

Supplementary Video S3 F- actin detection in *N. caninum*-infected cells (32 h p. i., 3D-reconstruction). PFA-fixed *N. caninum*-infected BUVEC were stained for F-actin (Phalloidin: green; the image corresponds to the one shown in Figure 7A). 3D reconstruction was performed by NIS element software built-in plugins. Background and signal levels were equally adjusted to illustrate the actin cytoskeleton in both non-infected and *N. caninum*-infected cells.
