# Corrigendum: *Neospora caninum* infection triggers S-phase arrest and alters nuclear characteristics in primary bovine endothelial host cells

**DOI:** 10.3389/fcell.2022.1025995

**Published:** 2022-09-20

**Authors:** Zahady D. Velásquez, Lisbeth Rojas-Barón, Camilo Larrazabal, Marcelo Salierno, Ulrich Gärtner, Learta Pervizaj-Oruqaj, Susanne Herold, Carlos Hermosilla, Anja Taubert

**Affiliations:** ^1^ Institute of Parasitology, Biomedical Research Center Seltersberg, Justus Liebig University Giessen, Giessen, Germany; ^2^ Centre for Developmental Neurobiology, MRC Centre for Neurodevelopmental Disorders, King’s College London, London, United Kingdom; ^3^ Institute of Anatomy and Cell Biology, Justus Liebig University Giessen, Giessen, Germany; ^4^ Department of Medicine V Internal Medicine Infectious Diseases and Infection Control Universities of Giessen and Marburg Lung Center (UGMLC) Member of the German Center for Lung Research (DZL) Justus-Liebig University Giessen, Giessen, Germany; ^5^ Institute for Lung Health (ILH), Giessen, Germany; ^6^ Excellence Cluster Cardipulmonary Institute (CPI), Giessen, Germany

**Keywords:** *Neospora caninum*, apicomplexan parasites, cell cycle arrest, nuclear lamina, actin-cap

In the published article, an author name was incorrectly written as Marcelo Saliermo. The correct spelling is “Marcelo Salierno”.

The authors apologize for this error and state that this does not change the scientific conclusions of the article in any way. The original article has been updated.

