# Supplementary figures and images for: Neospora caninum Infection Triggers S-phase Arrest and Alters Nuclear Characteristics in Primary Bovine Endothelial Host Cells
Source: Front Cell Dev Biol. 2022 Aug 5;10:946335. doi: 10.3389/fcell.2022.946335 (PMC9469085; doi:10.3389/fcell.2022.946335)

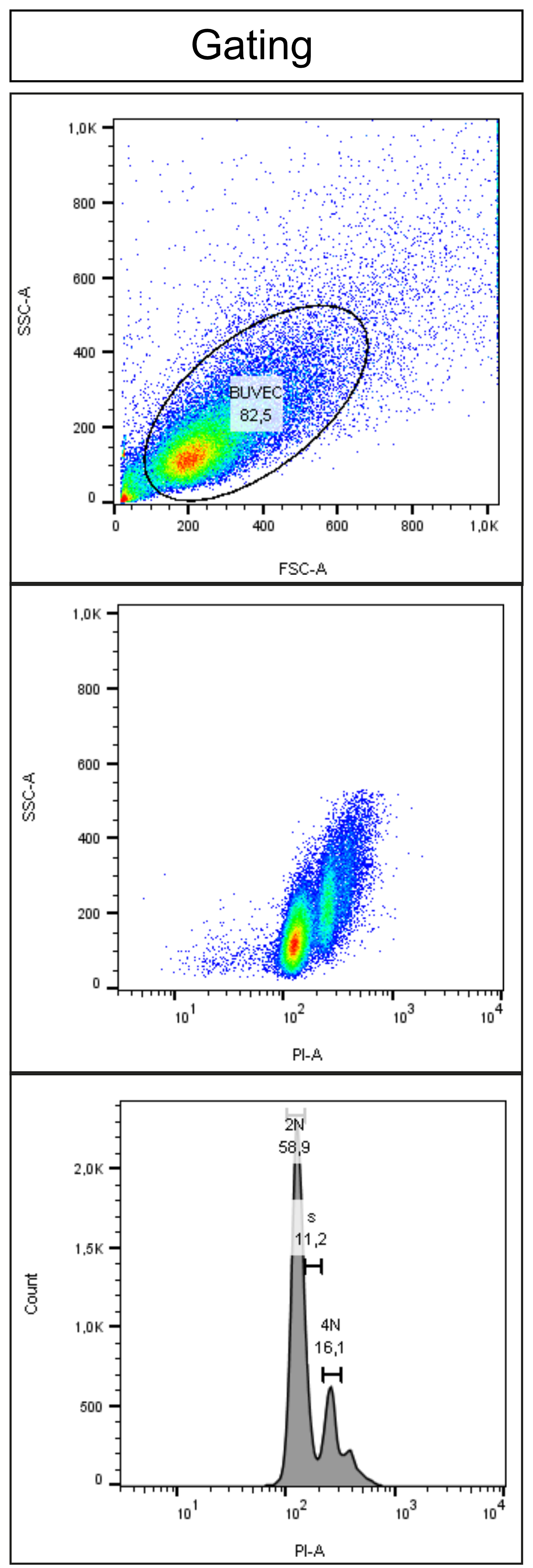

Supplement: Supplementary file 1 [file Image1.tiff]
